# Profiling recent medical graduates planning to pursue surgery, anesthesia and obstetrics in Brazil

**DOI:** 10.1186/s12909-019-1562-6

**Published:** 2019-05-08

**Authors:** Aline Gil Alves Guilloux, Jania A. Ramos, Isabelle Citron, Lina Roa, Julia Amundson, Benjamin B. Massenburg, Saurabh Saluja, Bruno Alonso Miotto, Nivaldo Alonso, Mario César Scheffer

**Affiliations:** 1 Faculdade de Medicina, da Universidade de São Paulo (FMUSP), Av. Dr. Arnaldo, 455 - Cerqueira César, São Paulo, SP 01246-903 Brazil; 2 Program in Global Surgery and Social Change, Harvard Medical School, 641 Huntington Ave, Boston, MA 02115 USA; 3 Duke University School of Medicine DUMC, Durham, NC 27710 USA; 4 Department of Obstetrics and Gynecology, University of Alberta, 116 St. and 85 Ave, Edmonton, AB T6G 2R3 Canada

**Keywords:** Surgical workforce, Medical education, Specialty selection, Global surgery, Anesthesia, Obstetrics

## Abstract

**Background:**

Lack of providers in surgery, anesthesia, and obstetrics (SAO) is a primary driver of limited surgical capacity worldwide. We aimed to identify predictors of entry into Surgery, Anesthesia, and Obstetrics and Gynecology (SAO) fields and preference of working in the public sector in Brazil which may help in profiling medical students for recruitment into these needed areas.

**Methods:**

A questionnaire was applied to all Brazilian medical graduates registered with a Board of Medicine from 2014 to 2015. Twenty-three characteristics were analyzed. Logistic regression was used to determine predictors’ influence on outcome.

**Results:**

There were 4601 (28.2%) responders to the survey, of which 40.5% (CI 34.7–46.5%) plan to enter SAO careers. Of the 23 characteristics analyzed, eight differed significantly between those who planned to work in SAO and those who did not. Of those eight characteristics, just three were significant predictors in the regression model: preference for working in the hospital setting, having spent more than 70% of their clinical years in practical activities, and valuing the substantial earning potential. These three factors explained only 6.3% of the variance in SAO preference. Within the graduates who preferred SAO careers, there were only two predictors for working in the public sector (“preparatory time before medical school” and valuing “prestige/status”).

**Conclusions:**

Factors affecting specialty and sector choice are multifaceted and difficult to predict. Future programs to fill provider gaps should identify methods other than medical student profiling to assure specialty and sector needs are met.

**Electronic supplementary material:**

The online version of this article (10.1186/s12909-019-1562-6) contains supplementary material, which is available to authorized users.

## Background

Over 90% of the population in Low- and Middle-Income Countries (LMIC) lack access to safe and affordable surgical care [1]. Low availability in the specialist surgical workforce, defined as fully trained physician surgeons, anesthetists, and obstetricians (SAO) combined, is one of the main drivers of the worldwide limited surgical capacity [1–3]. The Lancet Commission in Global Surgery recommends 20–40 SAO providers per 100,000 population as one of the indicators for a strong surgical system [2]. Brazil is an upper-middle income country with a population of almost 209 million people and an estimated average of 40 SAO providers per 100,000 capita [4–6]. However, there is an unequal distribution of providers across Brazil. The North Region only has 20.2 SAO providers and is at the verge of falling under the recommended density of SAO providers [2, 4]. In comparison, the more affluent South Region has 60.3 SAO providers per 100,000 [4]. As a result, 75.2% of all SAO providers in Brazil are working in regions where only 40.4% of the population lives [4–7].

Brazil has a free at point of care universal health care system, the *Sistema Único de Saúde* (SUS), and 28% of the population also has additional private health insurance coverage [7]. Physicians often work in both sectors, but the disproportionate distribution of physicians between the public and private health sectors further exacerbates the inequity of provider density. 21.6% of physicians work exclusively in the public sector while 26.9% work exclusively in the private sector despite serving only a minority of the population, with 51.5% working in both sectors [7]. Further compounding inequalities in SAO provision, the private sector tends to attract more of the specialists, with 68% of physicians working exclusively in the private sector being classified as specialists [5, 7]. Understanding and addressing the maldistribution of SAO providers, both geographically and within the private and public health sectors, is key in addressing the inequitable access to surgical care.

Mirroring the maldistribution of SAO providers, medical schools in Brazil are concentrated in the South and Southeast regions of the country, and in the capital cities rather than in rural areas. Throughout the country, 57% of all medical school positions are outside of capital cities, but in the North and Northeast regions the number of positions in rural areas decrease to 24.4 and 28.3% respectively [7]. Furthermore, due to severe financial pressures, the government of Brazil has announced austerity measures which include a 20-year freeze on public spending, including on education and healthcare [8]. To maintain quality of care, improved efficiency with how the current healthcare budget is spent is paramount. In Brazil, 35% of medical school positions available in 2017, as well as all residency positions, were funded by the federal government [9–11]. To ensure efficient spending on healthcare education, graduating physicians who have benefitted from federal sponsorship must go on to practice in the public system and in underserved specialties and underserved geographic areas.

The training of SAO providers in Brazil involves 6 years of medical school followed by three to 5 years of specialized rigorous residency training [12, 13]. Profiling of medical school entrants and graduates to select and train those who are likely to go into underserved SAO posts might represent an approach to ensure efficiency in training. In addition to characteristics for student selection, understanding which experiences during undergraduate training go on to influence the chances of medical students choosing SAO public sector careers can assist medical schools in adapting their curriculum towards producing more graduates in these underserved areas.

In this study we aimed to assess the personal characteristics and undergraduate experiences of recent medical graduates that predict entry into SAO careers, and to describe any characteristics that distinguish those who plan to work in the public instead of the private sector. This aims to establish the feasibility of targeted student recruitment or adaptations in undergraduate curriculums to improve recruitment to underserved SAO specialties and improve training efficiency in the era of austerity.

## Methods and analysis

### Survey

The survey used in this study consists of 104 multiple choice questions exploring demographics, personal characteristics, values and beliefs of the graduates, and aspects of their experiences in medical school (Additional file 1). The instrument also recorded the first and second choice of specialty that the graduates currently plan to enter for medical residency (Q58, Additional file 1). The survey was distributed electronically to 16,323 new medical school graduates previously registered with one of the 27 Regional Medical Councils (CRMs) of Brazil in 2015. Data was collected in two stages, first in Sao Paulo and then in other states, at the time of registration of new physicians at the CRMs. Distribution methods, survey development, inclusion and exclusion criteria, the survey instrument, adjustments, and validation have previously been described in detail [14].

### Definition of variables

Subgroup analysis for characteristics of graduates choosing a SAO specialty compared to other specialties was performed to examine the homogeneity of individual variables for the three specialties (Additional file 2). Based on the subgroup analysis and in accordance with the LCoGs definition of specialist surgical workforce, SAO careers were combined into one category for analysis. A SAO career was defined as selecting one of the following specialties as the first choice that the graduate plans to pursue for medical residency training: anesthesia, obstetrics and gynecology, cardiovascular surgery, hand surgery, head and neck surgery, GI surgery, general surgery, pediatric surgery, plastic surgery, thoracic surgery, vascular surgery, neurosurgery, orthopedic and traumatology, otorhinolaryngology, and urology (Q58, Additional file 1). Subgroup analysis for characteristics of graduates preferring SAO and also working in the public sector compared to the private sector was performed. Intent to work in the public or private sector was defined based on the response to the following question: “If pay, working conditions and number of hours were equivalent, would you choose to work in the public sector or in the private sector” (Q62, Additional file 1).

The survey used in this study was part of a large cohort study that evaluated 108 possible characteristics and undergraduate experiences. Of these, 23 characteristics were selected for comparison between those who plan to pursue SAO and non-SAO careers by authors M.S. and A.G., expert advisors to the Brazilian Government on medical workforce and faculty at the Sao Paulo School of Medicine, along with experts in the field of surgical public health policy and from previous literature in the field. These were selected based on literature review and the judgement and experiences of these experts as likely to be most relevant to decisions regarding SAO careers and thus most useful as selection criteria [15–21]. Of these characteristics, five were variables related to socio-demographic factors, eight related to experiences at medical school, and ten related to personal attitudes and values (Table 1).

**Table 1:** Characteristics of graduates who intend to pursue SAO careers compared to those who intend to pursue other specialties

Characteristic	Preference for SAO vs Other Specialties RR (95% CI)	Pearson’s P	Level of significance
Sociodemographic Factors			
Gender Male	1.358 (1.204–1.533)	< 0.000	^**^
Race White	0.914 (0.816–1.024)	0.117	
Family Income > 10x minimum wage	0.920 (0.851–0.995)	0.029	
Parent Edu Level beyond High School	0.934 (0.810–1.078)	0.32	
Other MD in family	0.945 (0.910–0.980)	0.005	^*^
Educational Experiences			
Public med school	0.921 (0.726–1.169)	0.481	
Took a year or less of a preparatory course for Medical School Entrance Exam	0.957 (0.905–1.011)	0.102	
Enrollment via Entrance Exam	0.943 (0.896–0.992)	0.028	
Participation in extracurriculars	1.310 (0.899–1.908)	0.105	
Volunteer work	1.043 (1.000–1.088)	0.047	
> 70% of clinical years spent in practical activities	0.862 (0.862–0.899)	< 0.000	^**^
Practical teaching of small/minor surgeries	0.933 (0.672–1.296)	0.664	
> =4 births on OBGYN with professor supervision	1.070 (0.964–1.188)	0.199	
Work Preferences			
Prefer to work in hospital	3.000 (2.309–3.897)	< 0.000	^**^
Prefer public practice	0.959 (0.834–1.103)	0.547	
Prefer flexible work day	0.889 (0.808–0.978)	0.023	
Desire CME opportunities	1.057 (1.007–1.110)	0.021	
Value interpersonal relations, human contact	0.883 (0.767–0.905)	< 0.000	^**^
Value the prestige/status	1.262 (1.139–1.399)	0.001	^**^
Value the interdisciplinary team	0.918 (0.884–0.955)	< 0.000	^**^
Value the (social) responsibility	1.242 (1.170–1.319)	< 0.000	^**^
Value substantial earning potential	1.234 (1.168–1.304)	< 0.000	^**^
Value liberty of action, professional autonomy	1.031 (0.905–1.174)	0.637	

^*^p < 0,009 and > 0.0022 – entered in the logistic model; ^**^p < 0,0022 – considered statistically associated and entered in the logistic model

All characteristic and experience variables were based on participant response to the multiple-choice answers given to each question in the questionnaire. Questions with multiple possible responses or Likert scale responses were collapsed as binary yes or no categorical variables.

### Statistical analysis

A univariate analysis was performed, with variables compared using relative risks (RR) and the chi-square test, with a Bonferroni adjusted level of significance of 0.0022. A forward selection logistic regression was performed using preference for SAO as the criterion variable and the individual characteristics and experiences as predictor variables. The first variable to enter into the model was that with lower association significance in the univariate analysis. It remained in the models if it’s significance level in the model was *p* < 0.0022. The next variables were then entered, one by one in the model, in order from the lower to the greater significance, up to *p* < 0.009 (four times the cutoff significance level). Variables with level of significance for association (chi-square) higher than 0.009 were not used in the analysis. When two predictor variables were associated with each other, only the one with lower significance level was entered in the model. McFadden Pseudo R^2^ was calculated as a measure of model fitting and estimation of total variance explained. Analysis were performed using IBM SPSS® Statistics version 21.

## Results

### Responses

**Fig. 1:**
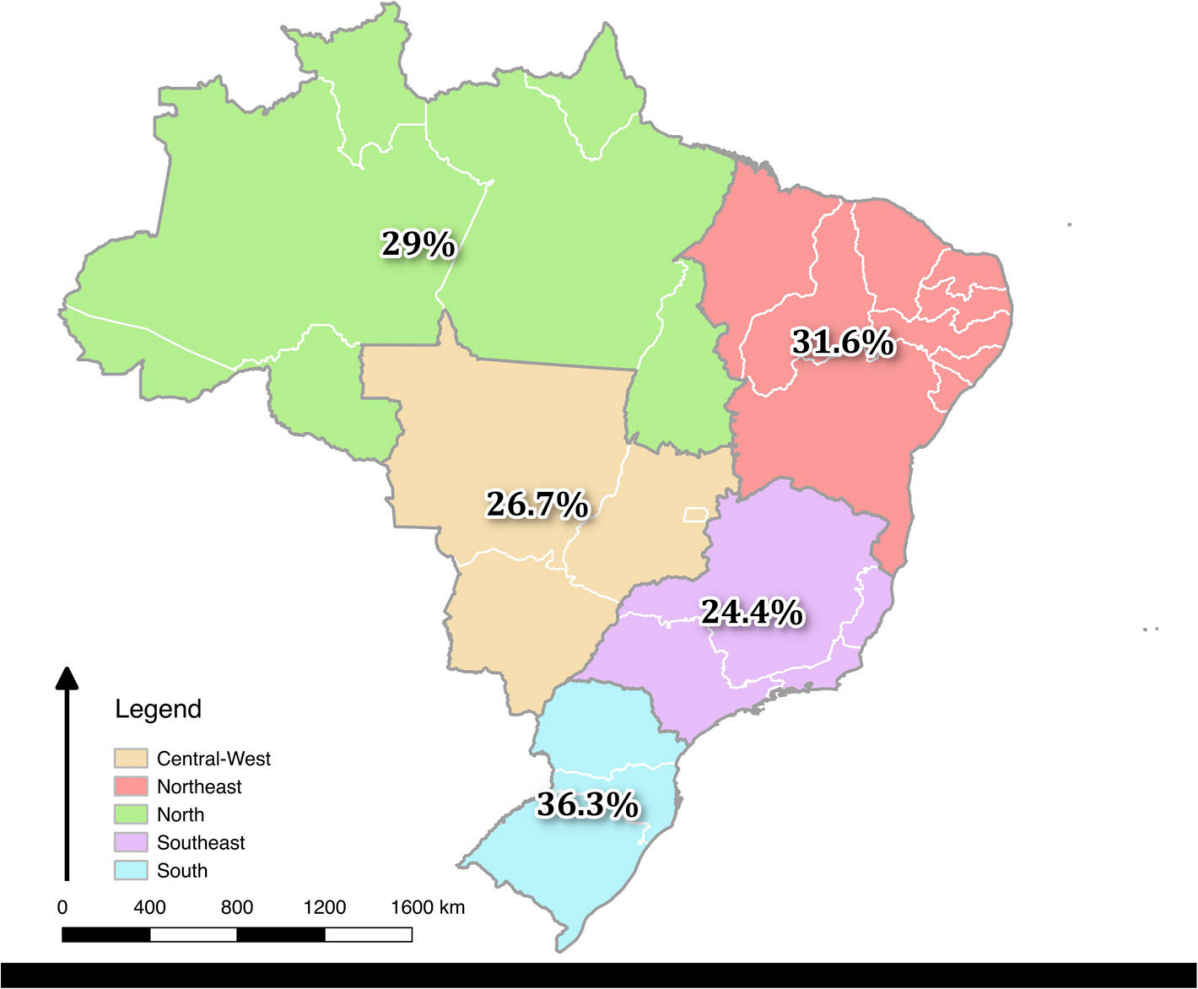
Map of Brazil, representing the response rate of the survey questionnaire per geographic region. This file has a figure that show the different response rate in each region and that was used as part of mathematical correction used to analyze all the data

The survey was distributed to 16,323 participants. There were 4601 eligible responses resulting in a response rate of 28.2%. There was some variation in response rate per region (Fig. 1) and the adequate representativeness was assured using mathematical adjustment for three factors: sex of the respondent, region of the country and funding of medical school (private or public).

The average age of participants is 27 years, 52.9% are female, 77.2% are white, and 57% come from families with a monthly income greater than ten times the minimum wage. More detailed demographics of respondents have been previously described, as well as comparisons of respondents’ demographics to the total population surveyed [14]. The subgroup analysis showed similar levels of significance between the three sub-specialties (Additional file 2). Of all survey respondents, 40.5% (CI 34.7–46.5%) selected that they intend to enter a Surgery, Anesthesia, or Obstetrics and Gynecology (SAO) career. Specifically, 8.6% (CI 4.5–15.8%) of all responders chose Gynecology and Obstetrics, 7.1% (CI 6.2–8.1%) chose anesthesia, and 8.8% (CI 6.2–12.2%) chose general surgery, with the remaining 15.8% choosing other surgical subspecialties.

### SAO vs other specialties

Of the 23 characteristics compared between graduates intending to pursue SAO careers and those intending to enter other specialties, eight were statistically different between the two groups (Table 1). Those responders who intended to pursue SAO careers were more likely to be male (*p* < 0.000) than those who intended to pursue other specialties. With regard to educational experience, those who intended to pursue SAO careers differed from other specialties in that they were less likely to have spent more than 70% of their clinical years in practical activities (*p* < 0.000). With regard to personal attitudes and values, those who intended to pursue SAO careers were more likely to prefer working in the hospital (*p* < 0.000), value the prestige or status of the profession (*p* = 0.001), value the responsibility of the profession (*p* < 0.000), and value the substantial earning potential (*p* < 0.000). They were less likely to value the interpersonal relations and human contact (*p* < 0.000) or the interdisciplinary team (*p* < 0.000). Those eight characteristics were selected to enter the logistic regression and a ninth one, having other MD in the family (*p* = 0,005), was also entered in the logistic regression (Additional file 3)

### Public vs Private

Results of the subgroup analysis assessing those who intended to pursue SAO careers in the public sector compared with those who intended to pursue SAO careers in the private sector if payments, work conditions and hours were equivalent, showed that those who would work in the public sector were less likely to have taken a year or less of preparatory courses for their medical school entrance exam and are less likely to value the prestige or status of the profession than those who would work in the private sector (Table 2). There were no other significant differences between the two groups across the other variables.

**Table 2:** Characteristics of those who intend to pursue SAO careers stratified by preference for working in the public vs the private sector

Characteristic	Preference for Public vs Private Sector RR (95% CI)	Pearson’s P	Level of Sig.
Sociodemographic Factors			
Gender Male	1.154 (0.838–1.591)	0.339	
Race White	0.955 (0.852–1.069)	0.419	
Family Income > 10xMinimum Wage	0.871 (0.786–0.965)	0.014	
Parent Educational Level Beyond High School	0.883 (0.746–1.044)	0.112	
Other MD in Family	1.064 (0.967–1.17)	0.172	
Educational Experiences			
Attended a Public Med School	1.382 (1.107–1.724)	0.004	
Took a year or less of a Preparatory Course for Medical School Entrance Exam	0.839 (0.768–0.916)	0.001	^**^
Enrollment via Entrance Exam	0.862 (0.654–1.135)	0.311	
Participated in Extra-Curricular Activities	1.257 (0.821–1.924)	0.255	
Participated in Volunteer Work	1.071 (0.848–1.353)	0.555	
> 70% of Clinical Years Spent in Practical Activities	0.999 (0.901–1.108)	0.980	
Received Practical Teaching of Small/Minor Surgeries	0.906 (0.72–1.139)	0.362	
> = 4 Births on OBGYN with Professor Supervision	1.022 (0.878–1.19)	0.766	
Work Preferences			
Prefer to Work in a Hospital	1.018 (0.685–1.514)	0.924	
Prefer a Flexible Work Day	0.964 (0.887–1.048)	0.355	
Desire CME Opportunities	1.022 (0.852–1.227)	0.806	
Value Interpersonal Relations, Human Contact	1.158 (0.951–1.41)	0.094	
Value the Prestige/Status	0.809 (0.747–0.876)	< 0.001	^**^
Value the Interdisciplinary Team	1.097 (0.967–1.244)	0.123	
Value the Responsibility	1.011 (0.958–1.067)	0.671	
Value Substantial Earning Potential	0.857 (0.722–1.016)	0.052	
Value Professional Autonomy	0.937 (0.758–1.158)	0.511	

^**^p < 0,0022 – as this characteristic was not modeled, we did not list the values that had *p* values in between 0.0022 and 0.009 that would be used in the model despite considered non-significant for our defined parameters

### Predicting choice

Of the nine characteristics tested in the regression model, only three were significant in the model of intention to pursue a SAO specialty: preference for working in the hospital setting, having spent less than 70% of their clinical years in practical activities, and valuing the substantial earning potential (Additional file 3). Collectively, these characteristics explain only 6.3% (McFadden Pseudo *R*^*2*^ = 0.063) of the preference for SAO.

## Discussion

This large study describes the characteristics of medical graduates selecting to go into SAO careers compared with other specialties, and of those, the characteristics of those intending to work in the public sector. This study highlights eight factors, mainly personal values and attitudes, which differentiate students choosing to pursue SAO specialties, and only two characteristics which differentiate those SAO professionals who would work in the public sector compared with the private sector. The combined predictive value of the characteristics for choosing SAO was just 6.3%, indicating that profiling and recruitment of medical students based on their likelihood to self-select into certain specialties may not be a viable strategy to improve medical training efficiency in this environment. Changes in training curriculums may also have only a marginal effect in changing chosen career paths.

The characteristic of students planning to pursue SAO careers identified in our study, including male gender, valuing the prestige and status of the profession, valuing the substantial earning potential, and valuing the responsibility, are similar to those identified by prior studies across different fields and different countries [15–21]. For example, in a study looking at career preferences in final year medical students in Kenya, Dossajee et al. found that the “perceived prestige of the specialty” was the main factor in choosing surgery as a career [21]. In a study in Canada by Wright et al. looking at factors influencing primary care choice, those choosing primary care were less likely to value “prestige” and “hospital setting” than those choosing specialty fields, both surgical and non-surgical [15]. These characteristics were also in agreement with those identified by two smaller Brazilian studies, one that surveyed 1st, 4th, and 5th year medical students in the Centro Universitário do Estado do Pará (Cesupa), and one that surveyed 5th and 6th year medical students in Salvador and Rio de Janeiro [22, 23]. Both of these studies found that “financial reasons” are a main driver of choosing specialties other than primary care, including surgical specialties.

Although we identified eight different characteristics associated with students planning to pursue SAO and non-SAO fields, only three were found to be significant predictors of SAO career choice in our regression model: preferring to work in a hospital setting, having spent less than 70% of clinical years in practical activities, and valuing the substantial earning potential of the profession. However, these three factors explained just 6.3% of the variance in SAO career choice preference. The analysis of proportion of variability explained by these variables is one of the strengths of this study. While other studies have highlighted a number of differing characteristics between groups of graduates, few have quantified the significance of these differences in making SAO career choices [15–23]. This poor predictive value suggest that although differences in characteristics may exist, they may not exert a true decision-making influence on students. Additionally, it is interesting to note that experiences in medical school also had little effect on the outcome of the choice of career in these students. These findings may point to the fact that adjustments in the undergraduate curriculum or profiling of medical school recruits may not be an effective way to improve training efficiency and matching of students into underserved specialties.

Instead, policies modulating extrinsic aspects of the job itself as opposed to intrinsic candidate specific attributes may be more successful. Examples of profession characteristics which have proved influential in the past include (1) the profile of the specialty, which includes the number of years of residency and the number of working hours [17, 24–26] (2) attractiveness of the labor market, especially social status and remuneration attributed to the specialty [17, 27], (3) ease of acceptance and career progression, for example, the number of medical residency positions, institutions and location of specialization [17, 20, 28, 29].

There are ongoing changes in Brazil affecting specialty variables described, in particular the number of residency positions. While specialties like Family and Community Medicine and Psychiatry have seen an increase in available positions, there has been minimal to no increase in positions for surgical specialties [9]. Furthermore, between 20 and 30% of residency positions in surgical fields remain unfilled each year [9]. In addition, incentives such as bonuses are being offered to certain specialties such as for those going into primary care in underserved rural areas, but not for SAO specialties [30]. In fact, within 2 years of existence, the program *Mais Médicos* instituted by the Brazilian government has recruited 18,425 physicians to fill primary care gaps by offering scholarships, increasing residency positions, and recruiting foreign physicians in primary care fields [30].

Limited access to emergency and essential surgical care, especially with regards to trauma and obstetric emergencies, in underserved areas of Brazil results in needless mortality and morbidity [31]. This highlights the key role of essential and emergency surgery as a component of primary care. Primary care doctors recruited as part of *Mais Médicos* incentives currently lack the skills required to perform basic emergency and essential surgical care and therefore cannot fill this gap. As a result, we suggest that SAO specialties should be included together with primary care specialties in future federal medical residency programs with similar incentivization of medical students to enter into SAO specialties serving the underserved public sector. Additional non-financial evidence-based incentives to attract SAO workers into the underserved public sector may also be needed such as offering housing accommodations and childcare for providers working as a SAO in the public sector in the most needed locations of the country and providing continuing medical education opportunities for SAO providers [17, 32, 33].

### Limitations

The study has a number of limitations. It is possible that despite the extensive number of aspects explored in the questionnaire there are other strong predictors of specialty choice which were not explored and therefore accurate prediction of specialty choice based on student characteristics is feasible. An additional limitation of the study is that the students were not asked the geographic location where they wanted to practice which may have given further information on drivers of geographic distribution. The response rate for the survey was 28.2% which although objectively low is quite high for a survey of this scale and mathematical adjustment was used to ensure representativeness of responses across demographics. Another limitation of the study is that recent graduates are describing their intended career choice or working sector, however not all will get a position in the desired residency and many may not work in the sector indicated. A follow up survey is planned in 10 years to assess this.

## Conclusion

This study suggests that there are differences between values and attitudes, demographic and undergraduate experiences of medical graduates who prefer SAO over other specialties, and those who would work in the public sector over the private sector. However, the profile of the upcoming workforce is unlikely to be significantly altered by profiling and targeting recruitment of certain medical students or by adjusting undergraduate experiences. Instead, to match the current workforce preferences to areas of need, characteristics of the jobs rather than the candidates should be targeted.

## Additional files

Additional file 1: Questionnaire. This file presents the English translation of the original questionnaire used to gather the data used in this paper. The questionnaire is similar to the Portuguese version including in its layout. (DOCX 67 kb)

Additional file 2: Characteristics analyzed in the Logistic Regression Model by each SAO specialty separately. This file presents a table with the characteristics of the students that chose SAO, separated by those who chose surgery, anesthesiology an obstetrics. (DOCX 32 kb)

Additional file 3: Regression Analysis for Predictors of Entry into SAO. This file presents, in a table, the models produced in the forward selection process, the coefficients and significance levels associated with each of them, reasons for keeping or removing all the variables and also the odds ratio for the final model for the paper. (DOCX 34 kb)

## Abbreviations

LMIC: Low and Middle-Income Country
SAO: Surgery, Anesthesia and Obstetrics
SUS: Sistema Único de Saúde
