# Supplementary material for: Profiling recent medical graduates planning to pursue surgery, anesthesia and obstetrics in Brazil
Source: BMC Med Educ. 2019 May 8;19:136. doi: 10.1186/s12909-019-1562-6 (PMC6505198; doi:10.1186/s12909-019-1562-6)
Supplement: Supplementary file 1 — Questionnaire. This file presents the English translation of the original questionnaire used to gather the data used in this paper. The questionnaire is similar to the Portuguese version including in its layout. (DOCX 67 kb) [file 12909_2019_1562_MOESM1_ESM.docx]

**Additional file 1: Questionnaire**

**BLOCK I GENERAL INFORMATION AND SOCIODEMOGRAPHIC**

1. Gender

Included on registration form.

1. Age

Included on registration form.

1. What's your nationality?

Included on registration form.

1. What is your marital status?

Included on registration form.

1. How many brothers/sisters do you have?

( ) None.

( ) One.

( ) Two.

( ) Three.

( ) Four or more.

1. What do you consider yourself?

( ) White

( ) Black

( ) Mixed / mulatto

( ) Yellow or of oriental origin

( ) Native or Indian origin

1. Who do you currently live with?

( ) With parents and / or other relatives.

( ) With spouse, partner, and / or child (ren).

( ) With Friends (sharing expenses)

( ) With colleagues in a university accommodation.

( ) Alone.

1. What is the monthly income range of your family? (Considering Family those who have lived together for more than 6 months)

( ) Up to 3x minimum wage

( ) 3x to 10x minimum wage

( ) 11x to 20x minimum wage

( ) 21x to 30x minimum wage

( ) More than 30x minimum wage

1. Check the situation below that best describes your case at the end of medical school

( ) Do not work and my expenses are funded by family.

( ) Work and receive help from family.

( ) Work and support myself.

( ) Work and contribute to the family's livelihood.

( ) Work and I am primarily responsible for the family's livelihood.

1. What is your father's level of education?

( ) No schooling.

( ) Primary school: from 1st to 4th grade.

( ) Primary school: 5th to 8th grade.

( ) High school.

( ) University.

1. What is your mother’s level of education?

( ) No schooling.

( ) Primary school: from 1st to 4th grade.

( ) Primary school: 5th to 8th grade.

( ) High school.

( ) University.

1. Are there other doctors in your family? (Consider just parents and / or siblings and spouse)

( ) Yes.

( ) No.

**BLOCOII - INITIAL TRAINING, ENTRANCE TO PROGRAM**

1. What kind of school did you attend in high school?

( ) All of the time in Public school.

( ) All of the time in private school.

( ) Most of the time (years) in a public school.

( ) Most of the time (years) in private school.

( ) Half in public school and half in private school.

1. Did you attend a preparatory course for the entrance exam of Medical School?

( ) Yes, I attended a preparatory course for 1 year or less.

( ) Yes, I attended a preparatory course for 2 years.

( ) Yes, I attended a preparatory course for 3 years or more.

( ) I did not attend a preparatory course.

1. What method did you use for entrance into Medical School?

( ) Traditional Vestibular.

( ) ESMS to complement the entrance examination scores.

( ) Sisu / MEC and or Quotas Law.

( ) Transfer from another institution.

( ) Revalidation.

( ) Other.

1. What kind of scholarship or funding grant did you receive for funding the expenses of medical school?

( ) I did not receive any scholarship or funding.

( ) Student Financing Fund - FIES.

( ) University for All Program -PROUNI.

( ) Full or Partial scholarship (or discounts on monthly fees) offered by the

institution.

( ) Full or partial scholarship offered by other entities (outside the institution).

( ) Other.

1. What are the main reasons that led you to choose medicine? (You can mark more than one answer)

( ) Interest in the study itself / intellectual challenge

( ) Interest in the doctor-patient relationship

( ) Scientific skills and ability to conduct research

( ) Prestige of the profession

( ) Potential compensation

( ) Per family influence or advice

( ) Possibility to carry out teaching

( ) Interest in the study of human body and disease

( ) Will make a difference in people's lives / do good

( ) Other

**BLOCK III - GRADUATION / COURSE ASSESSMENT**

1. The degree of satisfaction with your medical school?

( ) Very satisfied.

( ) Content.

( ) Neither satisfied nor dissatisfied.

( ) Dissatisfied.

( ) Very unsatisfied.

( ) I do not know how to answer.

1. How do you assess the level of demand of your courses, in regard to how they prepared you for the practice of medicine?

( ) Should have demanded a lot more of me

( ) Should have demanded a bit more of me.

( ) Demanded of me the right amount.

( ) Should have demanded a little less of me.

( ) Should have demanded a lot less of me.

1. To what extent do you agree with the statement: "I received enough preparation from my undergraduate courses to get a spot in medical residency."

( ) I totally agree.

( ) I agree in part.

( ) I totally disagree.

( ) I do not know.

1. What kind of academic activity were you involved in during your studies, in addition to those required? (You can mark more than one answer)

( ) Scientific research activities or research projects conducted by teachers.

( ) Monitoring activities.

( ) Community outreach activities or academic leagues.

( ) Artistic or cultural activities.

( ) Student leadership activities or Academic Center.

( ) Volunteer work

( ) Exchange with international schools.

( ) Other activities.

( ) No activity.

**BLOCK IV - GRADUATION: CURRICULUM, FACULTY, CONTENT**

1. How do you evaluate the curriculum of your studies?

( ) It was well articulated / integrated, there is clear link between the disciplines.

( ) It was relatively integrated, since the subjects are linked only by blocks or areas

of related knowledge.

( ) It was little integrated, as few disciplines are interconnected.

( ) No integration at all between disciplines.

( ) I do not know.

1. How was the availability of faculty members in the institution for extracurricular guidance?

( ) Everyone had availability.

( ) Most had availability.

( ) The minority had availability.

( ) None had availability.

1. Throughout your studies, how did you learn about the doctor-patient relationship?

( ) In most disciplines: through effective interaction with the patient.

( ) In most disciplines: while in the presence of the patient; the interaction with patient was exclusively by the teacher.

( ) In some disciplines: While in the presence of the patient; the patient interaction was exclusively by the teacher.

( ) In various disciplines: in general, without the patient present

( ) There was no particular concern of my school in this regard.

**ABOUT THE BASIC SCIENCE CURRICULUM**

1. How was most of the learning of the basic science curriculum?

( ) In groups, with teacher supervision, in the laboratory.

( ) Individually, with teaching supervision, in the laboratory.

( ) With demonstrations to the whole class, in the laboratory.

( ) Only theoretical in the classroom.

( ) None.

1. How would you evaluate the teaching supervision of educational programs of laboratory activities of the basic science curriculum?

( ) Fully adequate and sufficient.

( ) Proper and sufficient.

( ) Adequate, but not enough.

( ) Poor but sufficient.

( ) Inadequate and insufficient.

**ABOUT THE CLINICAL CURRICULUM / STAGE / INTERNSHIP**

1. Approximately what percentage of the clinical curriculum was aimed at practical activities?

( ) Less than 30%.

( ) 30 to 49%.

( ) 50 to 70%.

( ) More than 70%.

( ) I do not know how to evaluate.

1. Approximately what percentage of the clinical curriculum was performed within the hospital, with patients?

( ) Less than 30%.

( ) 30 to 49%.

( ) 50 to 70%.

( ) More than 70%.

( ) I do not know how to evaluate

1. Approximately what percentage of the clinical curriculum was performed in primary care / basic unit / clinic with patients?

( ) Less than 30%.

( ) 30 to 49%.

( ) 50 to 70%.

( ) More than 70%.

( ) I do not know how to evaluate.

1. What number of students per class, with a teacher, was more common in situations of patient care in practical activities?

( ) Less than four.

( ) From four to seven.

( ) Eight to Eleven.

( ) Twelve to fifteen.

( ) More than fifteen.

( ) I do not know

1. How was, in general, the smaller surgeries education (Outpatient surgery) carried out?

( ) Practical, with supervision at the school.

( ) Practical under supervision outside the school.

( ) Practical unsupervised at the school.

( ) Practical, unsupervised, outside the school.

( ) In my course there was no discipline that dealt with outpatient surgery.

1. Did your school have a skills lab (without the presence of patients) for learning procedures and invasive tests?

( ) Yes.

( ) No.

1. How was teaching in obstetrics carried out?

( ) Practical, with supervision at the school.

( ) Practical under supervision outside the school.

( ) Practical unsupervised at the school.

( ) Practical, unsupervised, outside the school.

( ) Only theoretical in the classroom.

1. In relation to the learning process in obstetrics, how many births did you do with the presence of the teacher?

( ) More than eleven.

( ) Eight to Eleven.

( ) From four to seven.

( ) From one to three.

( ) None.

1. What rating would you give the program in clinical areas?

( ) Adequate physical facilities; adequate and sufficient teaching supervision.

( ) Adequate physical facilities; adequate but insufficient teacher supervision.

( ) Adequate physical facilities; inadequate and insufficient teaching supervision.

( ) Inadequate physical facilities; inadequate teaching but sufficient supervision.

( ) Inadequate physical facilities; inadequate and insufficient teaching supervision.

1. Where did you complete your internship?

( ) Fully in the hospital / service of my school.

( ) Partly in the hospital / service of my school.

( ) Fully in a hospital arranged / accredited by my school.

( ) In hospital not connected / not accredited by my school.

1. What was, in your opinion, the greatest contribution of intern year?

( ) Technical and professional development.

( ) Knowledge of the labor market.

( ) Knowledge of new areas for graduates of the course.

( ) The reaffirmation of the professional choice made.

( ) The demonstration of the need for ongoing studies for effective professional practice.

**BLOCK V - graduate / ASPECTS OF TRAINING**

**With the following claims we want to know your agreement or disagreement on general aspects of your training in medicine.**

1. The program allowed me to learn what is the physician’s job.

( ) I totally agree.

( ) I agree in part.

( ) I totally disagree.

( ) I do not know

1. The program allowed me to understand what diseases are.

( ) I totally agree.

( ) I agree in part.

( ) I totally disagree.

( ) I do not know.

40. The program allowed me to understand the disease processes of people.

( ) I totally agree.

( ) I agree in part.

( ) I totally disagree.

( ) I do not know.

41. The program enhanced diagnosis through technologies.

( ) I totally agree.

( ) I agree in part.

( ) I totally disagree.

( ) I do not know.

42. The program enhanced diagnosis through conversations with the patient.

( ) I totally agree.

( ) I agree in part.

( ) I totally disagree.

( ) I do not know.

43. The organization and the volume of studies burdened students, even with possible negative impact on the health of students.

( ) I totally agree.

( ) I agree in part.

( ) I totally disagree.

( ) I do not know.

44. The training properly managed "how to deal with death."

( ) I totally agree.

( ) I agree in part.

( ) I totally disagree.

( ) I do not know.

45. The training properly managed sexuality.

( ) I totally agree.

( ) I agree in part.

( ) I totally disagree.

( ) I do not know.

46. The training properly managed conflict of interest, such as the relationship between doctors and pharmaceuticals and equipment industries.

( ) I totally agree.

( ) I agree in part.

( ) I totally disagree.

( ) I do not know.

47. The teaching of humanities and doctor patient relationship was part of my training.

( ) I totally agree.

( ) I agree in part.

( ) I totally disagree.

( ) I do not know.

48. In what situations, principally, can you say that you experienced / witnessed, in the program, any improper ethical conduct? (You can mark more than one answer).

( ) In relations with outpatients, ward, etc.

( ) In relations with family members of patients.

( ) In relations with the community and the general public.

( ) In relations with colleagues, staff, governing body.

( ) In decisions in the classroom.

( ) I experienced only appropriate ethical conduct.

**BLOCK VI - GRADUATION / ON COMPETENCE**

We would like to know how to assess your practical skills in some topics of the profession. Competence means your ownership and ability to use the knowledge obtained in medical school. In questions 49-55 are defined some skills that characterize the profession. Select to what extent each of the competencies was addressed in your graduation, using the following criteria:

|  | It was not addressed | It was partially addressed | It was fully addressed |
| --- | --- | --- | --- |
| 49. Clinical competence: doctors use scientific knowledge, technical skills and professional attitudes to treat their patients, as well as to diagnose clinical cases.  50. Communication competence: Doctors need to inform and make decisions; They need to know how to address and communicate with their patients, families and other health professionals. | ( )        ( ) | ( )        ( ) | ( )        ( ) |
| 51. Cooperation competence: Doctors work in partnership with other professionals that make up the services of health care. | ( ) | ( ) | ( ) |
| 52. The health system participant competence: As part of the health system, practices and decisions of physicians affect the resources, the effectiveness and efficiency of the health system, both for the National Health system as well as for the supplemental system (health insurance). | ( ) | ( ) | ( ) |
| 53. Competence of understanding of patient rights: doctors use their expertise and influence to promote the health and well-being of patients, the community and public health. | ( ) | ( ) | ( ) |
| 54. Competence of scientist, researcher and trainer: Doctors must learn continuously. For their scientific activities they contribute to create, disseminate, apply and use scientific knowledge. As trainers, they facilitate the training of students, patients, fellow doctors and others. | ( ) | ( ) | ( ) |
| 55. Competence to deal with ethical dilemmas: The practice of medicine is based on ethics and focused on the health and well-being of individuals and society. | ( ) | ( ) | ( ) |

**VII BLOCK PERSPECTIVE OF EXPERTISE**

56. What you plan to do now that you have graduated?

( ) Residency program at the same school where I finished the course

( ) Medical Residency Program in another school / institution

( ) Improvement / specialization in another kind of way, not a medical residency

( ) Immediate start of Medical practice

( ) Leave the country for work or specialization.

( ) Other activity

57. What is your role preferably?

( ) Clinic.

( ) Diagnostic methods.

( ) Surgery.

( ) Management, administration services.

( ) Teaching.

( ) Research.

( ) Other

58. Check what your 1st and 2nd choice for the completion of residency are. (Note that you must indicate only one specialty for first and another for the second option).

|  |  | FIRST | MONDAY |
| --- | --- | --- | --- |
| 1 | Acupuncture | ( ) | ( ) |
| 2 | Allergy and Immunology | ( ) | ( ) |
| 3 | Anesthesiology | ( ) | ( ) |
| 4 | Angiology | ( ) | ( ) |
| 5 | Oncology | ( ) | ( ) |
| 6 | Cardiology | ( ) | ( ) |
| 7 | Cardiovascular surgery | ( ) | ( ) |
| 8 | Hand Surgery | ( ) | ( ) |
| 9 | Head and Neck Surgery | ( ) | ( ) |
| 10 | Digestive System Surgery | ( ) | ( ) |
| 11 | General surgery | ( ) | ( ) |
| 12 | Pediatric surgery | ( ) | ( ) |
| 13 | Plastic surgery | ( ) | ( ) |
| 14 | Thoracic surgery | ( ) | ( ) |
| 15 | Vascular surgery | ( ) | ( ) |
| 16 | Medical clinic | ( ) | ( ) |
| 17 | Coloproctology | ( ) | ( ) |
| 18 | Dermatology | ( ) | ( ) |
| 19 | Endocrinology and Metabolism | ( ) | ( ) |
| 20 | Endoscopy | ( ) | ( ) |
| 21 | Gastroenterology | ( ) | ( ) |
| 22 | Medical genetics | ( ) | ( ) |
| 23 | Geriatrics | ( ) | ( ) |
| 24 | Gynecology and obstetrics | ( ) | ( ) |
| 25 | Hematology | ( ) | ( ) |
| 26 | Homeopathy | ( ) | ( ) |
| 27 | Infectious Diseases | ( ) | ( ) |
| 28 | Mastology | ( ) | ( ) |
| 29 | Family and Community Medicine | ( ) | ( ) |
| 30 | Occupational Medicine | ( ) | ( ) |
| 31 | Traffic Medicine | ( ) | ( ) |
| 32 | Sports medicine | ( ) | ( ) |
| 33 | Physical Medicine and Rehabilitation | ( ) | ( ) |
| 34 | Intensive medicine | ( ) | ( ) |
| 35 | Legal Medicine and Forensic Medicine | ( ) | ( ) |
| 36 | Nuclear medicine | ( ) | ( ) |
| 37 | Preventive and Social Medicine | ( ) | ( ) |
| 38 | Nephrology | ( ) | ( ) |
| 39 | Neurosurgery | ( ) | ( ) |
| 40 | Neurology | ( ) | ( ) |
| 41 | Nutrition | ( ) | ( ) |
| 42 | Ophthalmology | ( ) | ( ) |
| 43 | Orthopedics and Traumatology | ( ) | ( ) |
| 44 | Otorhinolaryngology | ( ) | ( ) |
| 45 | Pathology | ( ) | ( ) |
| 46 | Clinical Pathology / Laboratory Medicine | ( ) | ( ) |
| 47 | Pediatrics | ( ) | ( ) |
| 48 | Pneumonology | ( ) | ( ) |
| 49 | Psychiatry | ( ) | ( ) |
| 50 | Radiology and Diagnostic Imaging | ( ) | ( ) |
| 51 | Radiotherapy | ( ) | ( ) |
| 52 | Rheumatology | ( ) | ( ) |
| 53 | Urology | ( ) | ( ) |

**BLOCK VIII - perspective professional activity**

59. Thinking today, where do you intend to practice medicine?

( ) In the same city where I was born.

( ) In the same city where I completed my graduation.

( ) In the same city where I complete residency.

( ) In another place.

( ) I do not know how to answer.

60. What is the location of preference of your job of choice? (You can mark more than one answer)

( ) Hospital.

( ) Clinic.

( ) Private office.

( ) Basic health Unit.

( ) Family Medicine program.

( ) Clinical Laboratory.

( ) Pharmaceutical industry.

( ) University.

( ) Other.

61. Of the following aspects, which would be crucial for you to lay settle down in a city or workplace, after graduation and / or residency? (You can mark more than one answer)

( ) Work conditions.

( ) Salary, Compensation.

( ) Possibility of improvement, specialization.

( ) Safe environment, without violence.

( ) Professional recognition.

( ) Career path.

( ) Quality of life.

62. If pay, working conditions, and the number of work hours were equivalent, would you choose to work in the public sector or the private sector?

( ) Public sector.

( ) Private sector.

( ) Indifferent

**To reply to items 63 and 64: Add all possible positions / jobs, which would be the ideal monthly income: I-early in your career; and II- after five years of professional practice?**

63. I-START OF CAREER

( ) Up until R $ 8,000

( ) R $ 8,001 to R $ 12,000

( ) R $ 12,001 to R $ 16,000

( ) R $ 16,001 to $ 20,000

( ) R $ 20,001 to R $ 24,000

( ) R $ 24,001 or more

( ) I do not know how to answer

64. II- AFTER 5 YEARS OF WORK

( ) Up until R $ 8,000

( ) R $ 8,001 to R $ 12,000

( ) R $ 12,001 to R $ 16,000

( ) R $ 16,001 to $ 20,000

( ) R $ 20,001 to R $ 24,000

( ) R $ 24,001 or more

( ) I do not know how to answer.

65. If you could choose, what would be your preferred method of compensation for the exercise of your medical profession?

( ) Monthly salary.

( ) Compensation per hour worked.

( ) Compensation for number of procedures.

( ) Compensation for number of patients.

( ) Pay for performance (as stipulated by my performance goal).

( ) Compensation for contract.

( ) Mixed income (of several types).

( ) Indifferent.

66. At what age would you choose to stop exercising your occupation, assuming you are in good health and free to choose?

( ) Between 50 and 60

( ) Between 60 and 70 years

( ) Between 70 and 80 years

( ) After 80 years

( ) Do not know

**BLOCK IX -about the profession and career**

67. For you, what are the most important factors for a satisfying and rewarding professional practice? (You can answer more than one option)

( ) The ability to achieve a balance between professional and personal life.

( ) Time / flexible working hours.

( ) Have sufficient technical expertise to meet the health needs of my patients.

( ) Exercise only one specialty.

( ) Availability of resources for development and continuing education.

( ) Ability to conduct research.

( ) Ability to exercise education.

( ) Having a health system that gives appropriate assistance to my patients.

( ) I do not know how to answer.

68. Of the following characteristics, which you appreciate most in the medical profession? (You can mark more than one answer)

( ) Human contact, interpersonal relationships.

( ) The need to seek constant improvement.

( ) The prestige / status that the profession has in society.

( ) The work of analysis and reflection that is required for diagnosis.

( ) The possibility to treat, to heal.

( ) The liberal character and autonomy of the profession.

( ) Social responsibility of the physician, regardless of the compensation gains.

( ) The work of an interdisciplinary team.

( ) The responsibilities.

( ) Research work.

( ) Substantial compensation.

( ) Others.

69. What are the obstacles that seem most relevant in relation to medicine in Brazil today? (You can mark more than one answer)

( ) Freedom of insufficient action / interference in professional autonomy.

( ) Poor working conditions.

( ) Excessive Hours

( ) The need to have multiple positions to make an income.

( ) Loss of income without reduction of the workload.

( ) Risk of falling into a routine.

( ) Requirements of the point of view of physical strength

( ) Climate of tension and rivalry in the work environment.

( ) Personal life absorbed by the profession / difficulty of reconciling personal life with professional life.

( ) Intermediation of the profession by health insurance plans

( ) Lack of jobs and low pay in the SUS.

( ) Elitism and detachment of the profession from the real needs of the population.

( ) Others.

**BLOCK X -About HEALTH SYSTEM**

**Here we want to know your views on some aspects of the Brazilian health system and the role of the physician in this system.**

70. Brazil should ensure health as a right of all and duty of the State, through universal coverage and equitable public health.

( ) I totally agree.

( ) I agree in part.

( ) I totally Disagree.

( ) I do not know.

71. To ensure adequate public funding and expand the SUS is the best solution for the Brazilian health system.

( ) I totally agree.

( ) I agree in part.

( ) I totally disagree.

( ) I do not know.

72. Increasing access of the population to health plans and health insurance is the best solution for the Brazilian health system.

( ) I totally agree.

( ) I agree in part.

( ) I totally disagree.

( ) I do not know

73. SUS should only be targeted to low-income or unemployed citizens.

( ) I totally agree.

( ) I agree in part.

( ) I totally disagree.

( ) I do not know.

74. Those who can should pay directly for health through the purchase of medications, health insurance plans or private consultations.

( ) I totally agree.

( ) I agree in part.

( ) I totally disagree.

( ) I do not know.

75. A major health problem in Brazil is the lack of adequate public funding.

( ) I totally agree.

( ) I agree in part.

( ) I totally disagree.

( ) I do not know

76. A major health problem in Brazil is the mismanagement and disorganization of the health system.

( ) I totally agree.

( ) I agree in part.

( ) I totally disagree.

( ) I do not know.

77. The functioning of the health system today does not allow for comprehensive care that is adapted to the patient's needs.

( ) I totally agree.

( ) I agree in part.

( ) I totally disagree.

( ) I do not know.

78. The doctor who works in the NHS does not have the time required for a relationship with the patient.

( ) I totally agree.

( ) I agree in part.

( ) I totally disagree.

( ) I do not know.

79. The doctor who serves health insurance plans does not have the time for a good relationship with the patient.

( ) I totally agree.

( ) I agree in part.

( ) I totally disagree.

( ) I do not know.

80. The payment per procedure and for medical actions prevents a more humanized care to the patient.

( ) I totally agree.

( ) I agree in part.

( ) I totally disagree.

( ) I do not know.

81. The overuse of technologies makes it difficult for the doctor to have a closer relationship with patients.

( ) I totally agree.

( ) I agree in part.

( ) I totally disagree.

( ) I do not know.

82. Medical work is now undervalued.

( ) I totally agree.

( ) I agree in part.

( ) I totally disagree.

( ) I do not know.

**BLOCK XI to -about Ethics / BIOETHICAL**

**For questions numbers 83-103, we would like to know your opinion on topics that are being debated in society. So, next to each statement indicate what is your position on the following criteria:**

**On abortion:**

It should be performed:

|  | I agree | Disagree | I prefer not to answer |
| --- | --- | --- | --- |
| 83. In the event of rape. | ( ) | ( ) | ( ) |
| 84. In case of risk to health. | ( ) | ( ) | ( ) |
| 85. In case of risk to the mother's life. | ( ) | ( ) | ( ) |
| 86. In cases of malformation of the fetus. | ( ) | ( ) | ( ) |
| 87. Only up to 12 weeks of gestation. | ( ) | ( ) | ( ) |
| 88. Under any circumstances. | ( ) | ( ) | ( ) |

**About the end of life:**

The doctor:

|  | I agree | disagree | I prefer not to answer |
| --- | --- | --- | --- |
| 89. Should limit or suspend procedures and patient treatments that prolong the life of the patient who is terminally ill or incurable. | ( ) | ( ) | ( ) |
| 90. Must limit or suspend procedures and treatments, respecting the wishes expressed by the patient. | ( ) | ( ) | ( ) |
| 91. Must abide by the "living will" the patient (advance directives of will) about the care and treatment he or she wishes to receive. | ( ) | ( ) | ( ) |
| 92. Must make use of all technological resources that can ensure the patient's life, even those terminally ill. | ( ) | ( ) | ( ) |

**On assisted reproduction**

Acceptable procedures:

|  | I agree | disagree | I prefer not to answer |
| --- | --- | --- | --- |
| 93. Temporary donation of a uterus ( "surrogate"), subject to the agreement and consent of the parties. | ( ) | ( ) | ( ) |
| 94. Temporary donation of a uterus ( "surrogacy"), only when there is a proven consanguineous relationship. | ( ) | ( ) | ( ) |
| 95. The use of assisted reproduction techniques in the case of homosexual relationships. | ( ) | ( ) | ( ) |
| 96. The use of assisted reproduction techniques in the case of relationships between unmarried people. | ( ) | ( ) | ( ) |
| 97. Disposal of cryopreserved embryos, if that is the will of patients. | ( ) | ( ) | ( ) |
| 98. The use of embryos in research. | ( ) | ( ) | ( ) |

**On the doctor's relationship with the pharmaceutical and equipment/supplies industry:**

|  | I agree | disagree | I prefer not to answer |
| --- | --- | --- | --- |
| 99. Medical conferences, publications and continuing education programs can be financed by the industry. | ( ) | ( ) | ( ) |
| 100. The doctor can receive gifts of small value and travel funding for conferences | ( ) | ( ) | ( ) |
| 101. The industry representative visit influences the doctor's prescription practices. | ( ) | ( ) | ( ) |
| 102. It is correct for the industry to finance "cervejadas", barbecues and cocktails for students and residents. | ( ) | ( ) | ( ) |
| 103. The physician should be prohibited from linking medical prescription practices to the receipt of material benefits or financial support. | ( ) | ( ) | ( ) |

104. What should be the priority actions of the Regional and Federal Councils of Medicine? (You can mark up to three options).

( ) Perform trial of doctors who commit ethical violations.

( ) Disseminate technical and medical ethics standards.

( ) Monitor the doctor's working conditions.

( ) Act for better remuneration (compensation) of doctors.

( ) To undertake actions aimed at improving the Unified Health System (SUS).

( ) Regulate the relationship of health plans with doctors.

( ) Contribute to the improvement of medical education and medical residency.

( ) Sponsor free programs of continuing education.

( ) To undertake actions for the implementation of the medical career in the SUS.

( ) Facilitate access complaints channels of the population to CRMs.

( ) Other actions.
