# Supplementary material for: Profiling recent medical graduates planning to pursue surgery, anesthesia and obstetrics in Brazil
Source: BMC Med Educ. 2019 May 8;19:136. doi: 10.1186/s12909-019-1562-6 (PMC6505198; doi:10.1186/s12909-019-1562-6)
Supplement: Supplementary file 2 — Characteristics analyzed in the Logistic Regression Model by each SAO specialty separately. This file presents a table with the characteristics of the students that chose SAO, separated by those who chose surgery, anesthesiology an obstetrics. (DOCX 32 kb) [file 12909_2019_1562_MOESM2_ESM.docx]

**Additional file 2: Characteristics analyzed in the Logistic Regression Model by each SAO specialty separately**

| **Characteristic** | **Preference for Surgery vs Other Specialties RR (95% CI)** | **Pearson’s P** | **Level of Sig.** | **Preference for Anesthesiology vs Other Specialties RR (95% CI)** | **Pearson’s P** | **Level of Sig.** | **Preference for Obstetrics vs Other Specialties RR (95% CI)** | **Pearson’s P** | **Level of Sig.** |
| --- | --- | --- | --- | --- | --- | --- | --- | --- | --- |
| **Sociodemographic Factors** | | | | | | | | | |
| *Gender Male* | 2.193 (1.896 - 2.537) | 0.000 | ** | 1.394 (1.234 - 1.574) | 0.000 | ** | 0.182 (0.139 - 0.24) | 0.000 | ** |
| *Race White* | 0.877 (0.754 - 1.02) | 0.064 |  | 1.215 (0.85 - 1.738) | 0.264 |  | 0.833 (0.593 - 1.171) | 0.284 |  |
| *Family Income >10x minimum wage* | 0.902 (0.799 - 1.018) | 0.068 |  | 1.038 (0.876 - 1.23) | 0.652 |  | 0.881 (0.788 - 0.985) | 0.028 |  |
| *Parent Edu Level beyond High School* | 0.857 (0.649 - 1.132) | 0.234 |  | 0.875 (0.717 - 1.068) | 0.178 |  | 1.32 (1.063 - 1.64) | 0.012 |  |
| *Other MD in family* | 0.884 (0.83 - 0.942) | 0.001 | ** | 1.431 (1.081 - 1.896) | 0.014 |  | 0.784 (0.653 - 0.941) | 0.017 |  |
| **Educational Experiences** | | | | | | | | | |
| *Public med school* | 0.961 (0.521 - 1.772) | 0.893 |  | 1.167 (0.874 - 1.557) | 0.278 |  | 0.637 (0.242 - 1.68) | 0.340 |  |
| *Took a year or less of a preparatory course for Medical School Entrance Exam* | 0.996 (0.908 - 1.093) | 0.933 |  | 0.994 (0.828 - 1.193) | 0.942 |  | 0.825 (0.598 - 1.137) | 0.212 |  |
| *Enrollment via Entrance Exam* | 0.824 (0.621 - 1.095) | 0.144 |  | 0.777 (0.549 - 1.1) | 0.148 |  | 2.061 (1.093 - 3.888) | 0.027 |  |
| *Participation in extracurriculars* | 1.426 (0.975 - 2.084) | 0.037 |  | 0.879 (0.384 - 2.011) | 0.750 |  | 1.567 (1.123 - 2.186) | 0.008 | * |
| *Volunteer work* | 1.025 (0.935 - 1.124) | 0.589 |  | 0.933 (0.815 - 1.069) | 0.299 |  | 1.201 (1.003 - 1.439) | 0.057 |  |
| *>70% of clinical years spent in practical activities* | 0.794 (0.685 - 0.92) | 0.001 | ** | 0.762 (0.526 - 1.104) | 0.142 |  | 1.2 (0.855 - 1.684) | 0.260 |  |
| *Practical teaching of small/minor surgeries* | 1.032 (0.787 - 1.354) | 0.813 |  | 0.861 (0.52 - 1.424) | 0.540 |  | 0.765 (0.593 - 0.986) | 0.050 |  |
| *>=4 births on OBGYN with professor supervision* | 1.057 (0.973 - 1.149) | 0.201 |  | 0.891 (0.75 - 1.058) | 0.176 |  | 1.291 (0.941 - 1.77) | 0.093 |  |
| **Work Preferences** | | | | | | | | | |
| *Prefer to work in hospital* | 4.203 (2.356 - 7.5) | 0.000 | ** | 4.583 (1.632 - 12.871) | 0.002 | * | 1.333 (1.049 - 1.695) | 0.026 |  |
| *Prefer public practice* | 0.974 (0.76 - 1.248) | 0.826 |  | 0.74 (0.611 - 0.897) | 0.003 | * | 1.133 (0.943 - 1.363) | 0.162 |  |
| *Prefer flexible work day* | 0.848 (0.738 - 0.974) | 0.030 |  | 1.211 (0.844 - 1.738) | 0.277 |  | 0.802 (0.666 - 0.967) | 0.028 |  |
| *Desire CME opportunities* | 1.076 (0.922 - 1.256) | 0.359 |  | 0.885 (0.555 - 1.411) | 0.589 |  | 1.153 (0.953 - 1.394) | 0.141 |  |
| *Value interpersonal relations, human contact* | 0.814 (0.737 - 0.9) | 0.000 | ** | 0.441 (0.342 - 0.569) | 0.000 | ** | 1.772 (1.411 - 2.224) | 0.000 | ** |
| *Value the prestige/status* | 1.304 (1.068 - 1.592) | 0.032 |  | 1.312 (1.052 - 1.637) | 0.018 |  | 1.111 (0.718 - 1.719) | 0.625 |  |
| *Value the interdisciplinary team* | 0.848 (0.743 - 0.968) | 0.009 | * | 0.88 (0.645 - 1.199) | 0.397 |  | 1.165 (0.997 - 1.362) | 0.051 |  |
| *Value the (social) responsibility* | 1.35 (1.194 - 1.526) | 0.000 | ** | 1.297 (1.026 - 1.638) | 0.030 |  | 0.92 (0.774 - 1.094) | 0.327 |  |
| *Value substantial earning potential* | 1.262 (1.085 - 1.468) | 0.018 |  | 1.075 (0.842 - 1.371) | 0.544 |  | 1.288 (0.975 - 1.702) | 0.086 |  |
| *Value liberty of action, professional autonomy* | 0.987 (0.77 - 1.265) | 0.911 |  | 1.133 (0.705 - 1.821) | 0.588 |  | 1.078 (0.952 - 1.221) | 0.222 |  |
