# Supplementary material for: Profiling recent medical graduates planning to pursue surgery, anesthesia and obstetrics in Brazil
Source: BMC Med Educ. 2019 May 8;19:136. doi: 10.1186/s12909-019-1562-6 (PMC6505198; doi:10.1186/s12909-019-1562-6)
Supplement: Supplementary file 3 — Regression Analysis for Predictors of Entry into SAO. This file presents, in a table, the models produced in the forward selection process, the coefficients and significance levels associated with each of them, reasons for keeping or removing all the variables and also the odds ratio for the final model for the paper. (DOCX 34 kb) [file 12909_2019_1562_MOESM3_ESM.docx]

**Additional file 3: Regression Analysis for Predictors of Entry into SAO**

| **Prefer SAO*** | Model 1 | | Model 2 | | Model 3 | | **Model 4 - final** | | **OR of final model** | Model 5 | | Model 6 | | Dropped variables^1^ | |
| --- | --- | --- | --- | --- | --- | --- | --- | --- | --- | --- | --- | --- | --- | --- | --- |
|  | **ß**  **(95%CI)** | **p** | **ß**  **(95%CI)** | **p** | **ß**  **(95%CI)** | **p** | **ß**  **(95%CI)** | **p** |  | **ß**  **(95%CI)** | **p** | **ß**  **(95%CI)** | **p** | **ß**  **(95%CI)** | **p** |
| Revere the (social) Responsibility* | 0.045  (-0,121; 0.212) | 0.576 |  |  |  |  |  |  |  |  |  |  |  |  |  |
| **Prefer to work in a hospital setting*^a^** |  |  | 1.565  (1.282; 1.847) | < 0.001 | 1.592  (1.347; 1.837) | < 0.001 | **1.616**  **(1.386; 1.846)** | **< 0.001** | **5.032 (3.997; 6.334)** | 1.622 (1.400; 1.845) | < 0.001 | 1.614  (1.386; 1.842) | < 0.001 |  |  |
| **> 70% of their clinical years in practical activities*** |  |  |  |  | -0.250  (-0.317; -0.182) | < 0.001 | **-0.235**  **(-0.309; -0.160)** | **< 0.001** | **0.791 (0.734; 0.852)** | -0.234 (-0.309; -0.158) | < 0.001 | -0.237  (-0.313; -0.161) | < 0.001 |  |  |
| **Value the substantial earning potential*^b^** |  |  |  |  |  |  | **0.433**  **(0.286; 0.579** | **< 0.001** | **1.541 (1.331; 1.785** | 0.441 (0.290; 0.592) | < 0.001 | 0.429  (0.283; 0.575) | < 0.001 |  |  |
| Gender male^# a b^ |  |  |  |  |  |  |  |  |  |  |  |  |  | 1.069 (0.842; 1.296) | < 0.001 |
| Revere interpersonal relations, human contact*^b^ |  |  |  |  |  |  |  |  |  |  |  |  |  | -0.277 (-0.396; -0.158) | < 0.001 |
| Revere the interdisciplinary team* |  |  |  |  |  |  |  |  |  | -0.197 (-0.325; -0.070) | 0.004 |  |  |  |  |
| Revere the prestige/status*^b^ |  |  |  |  |  |  |  |  |  |  |  |  |  | 0.362 (0.033; 0.692) | 0.033 |
| Other MD in family* |  |  |  |  |  |  |  |  |  |  |  | -0.032  (-0.121; 0.057) | 0.463 |  |  |
| **McFadden Pseudo R^2^** | <0.001 | | 0.055 | | 0.060 | | 0.063 | | | 0.064 | | 0.063 | |  | |
| *Reference category: ‘No’; ^#^Reference category: ‘Female’  ^a^ Variables associates with “prefer to work in a hospital setting” that is already in the model, thus were not entered in the model.  ^b^ Variables associated with “value the substantial earning potential^”^ that is already in the model, thus were not entered in the model.  The black shade in the table represent variables that were either dropped from the analysis or were not entered for their association with other variables already in the model  ^1^The variables that were not entered in the model were tested one by one in empty models and their parameters are presented in the last column of the table. | | | | | | | | | | | | | | | |
